# Facing the faceless patients – the emerging challenges of identity fraud in general surgery: A case series

**DOI:** 10.1016/j.ijscr.2018.11.041

**Published:** 2018-11-22

**Authors:** Jason Cui, Rasika Hendahewa

**Affiliations:** General Surgical Department, Caboolture Hospital, Caboolture, QLD, 4510, Australia

**Keywords:** General surgery, Identity fraud, Laparoscopy, Health care system

## Abstract

**Introduction:**

This case series highlights the challenges faced in general surgery when encountering patients who use multiple false identities and provide unreliable histories.

**Presentation of cases:**

We present two patients both with a background of mental health disorders and previous abdominal surgeries. After extensive workup with one patient almost proceeding to surgery, it was noted that these patients have had multiple presentations under different aliases to multiple different hospitals and had given inconsistent medical and surgical histories.

**Discussion:**

In such patients, history and clinical examinations are unreliable. Medical records are also not helpful given the patients’ multiple aliases. Particularly in surgery, inaccurate knowledge of the patients’ previous operative details can lead to significant harms. Extensive investigations are usually performed in assessing these patients, resulting in excessive radiation exposure and incurring significant costs to the health system. It is also important to recognise the potential legal repercussions and harms to the patients who have had their identities stolen.

**Conclusion:**

Identity fraud poses significant challenges to patient care, vigilance from clinicians and implementing strategies to reduce system error can help to limit harms to the patient and the health care system.

## Highlights

• Identity fraud is becoming a more recognizable challenge, particularly with the introduction of electronic medical records.
• Particularly in surgery, identity fraud can result in significant harm to patients and burden to the health care system.
• Clinical vigilance and implementation of strategies to reduce system error can reduce the negative impact of identity fraud.

## 1 Introduction

This case report is in line with the PROCESS criteria [6].

In Australia, all permanent residents are eligible for a Medicare Card with a unique number. Upon presentation, it allows patients access to the public hospitals free of charge. In Queensland State, majority of the hospitals are linked to an electronic system which identifies patients based on their Medicare number. It records patients’ hospital encounters and summaries of their inpatient managements.

In most cases, the system allows for accurate identification of patients and access to their medical records. It also flags patients with high volume presentations in case a pre-planned management has been implemented. However, as both the Medicare Card and the electronic medical systemic lack photo identification of the patients, there are cases where a record is created either with a falsified or stolen identity.

The workup and management of these patients can be complex. Harm to patients can result from excessive or invasive investigations or operative interventions based on inaccurate histories. It also causes significant strain on the health care system and cause harm to the patients who have had their identities stolen.

## 2 Case presentation

### 2.1 Case 1

A 21-year-old female with no prior encounters presented to the emergency department with right iliac fossa pain and a history concerning for appendicitis. She reported a background of having been involved in a serious motor vehicle accident overseas 3 months ago which resulted in the death of her sister. She has also required an emergency laparotomy and bowel resection with prolonged ICU stay.

On examination, a midline laparotomy scar was evident and the patient was tender over the right iliac fossa with no features of peritonism. Her white cell count was equivocal and C-reactive protein unremarkable. A pelvic ultrasound scan was performed which was unsuccessful in identifying the appendix. On CT, the appendix was again not visualised, however there were no secondary signs of appendicitis.

The patient was admitted for observation and serial assessment, after 24 h of admission, it was noted that patient required significant amounts of opioids with no clinical evidence of appendicitis nor biochemical evidence to suggest inflammation. The acute pain service as well as the drug dependence teams were consulted. After extensive investigation into her background, it was discovered that she had 23 different aliases and countless hospital presentations with drug seeking behaviours. Her overseas injuries were falsified and her sister was also alive and well. Shortly after the discovery, the patient discharged against medical advice.

### 2.2 Case 2

A female patient in her 30 s presented to the emergency department with a classical history of appendicitis. She reported a background of previous penetrating injuries to her abdomen and having had a splenectomy secondary to trauma. There was also self-reported anxiety, depression and post-traumatic stress disorder. There were no remarkable recent hospital presentations on reviewing her electronic record.

After assessment by two general surgeons, it was deemed that appendicectomy was indicated. The patient has initially agreed to undergo appendicectomy, however she refused to sign the consent form and stated that she has extreme anxiety and phobia of the operating theatre. She was commenced on antibiotics as a temporary measure whilst consult from the psychiatric and anaesthetic team were obtained. After extensive discussions and exploration of options, it was decided with the patient to undergo sedated on the ward by the anaesthetist prior to transfer to the theatre for appendicectomy.

However, before her planned operation, the patient has left the hospital without giving notice. The hospital coordinators attempted to recall the patient and discovered that the identity the patient used belonged to a different person who resides in a different region. It was also by chance that upon discussing her at our meeting, a surgeon who has previously worked at a different centre has recognised her and advised that this patient has had multiple presentations to other centres with the same.

## 3 Discussion

Providing surgical care to patients with co-morbid mental health disorders has been an ongoing challenge. A number of studies have found a significant increase in peri-operative complications in patients with mental health disorders such as depression, anxiety and schizophrenia [[1], [2], [3]]. As discussed by Bennett [4], this association is possibly a result of the higher incidences of smoking, hypertension, obesity and drug abuse in this particular population, furthermore, there is the tendency for this group to cause disruptive behaviours which also may lead to isolation and reduced attendance from the treating teams. However, there has been no studies which focus on the detrimental effects of patients with false identities in general surgery.

Through these two case examples, multiple challenges have been highlighted. Firstly, the inaccurate reporting of one’s surgical history can complicate surgical planning and result in unnecessary operations, putting patients at increased peri-operative risks. In the case of the second patient, if she did proceed to having an appendicectomy, it may be reasonable to perform it as an open procedure instead of a laparoscopic procedure to avoid the risks of port related injuries given her previous laparotomy. The scar would also provide information regarding her surgical history if she has subsequent presentations. Harms to these patients may also result from excessive investigations and over-treatment. These include the excessive radiation exposure from medical imaging as well as the excessive prescribing of opioids in patients with drug seeking behaviours. Conversely, there is also a risk for these patients to be neglected once they have been labelled as in pursuit of secondary gains. It may be difficult to differentiate the “true symptoms” from the fabricated ones and delays in recognition and management could result in significant harms. It is also important to note the burdens on the health care system, these complex patients require more medical attention and the cost in their investigations and managements, particularly if they present multiple times. Lastly, the patients who have had their identities misused or stolen will also be significantly affected. Their medical records may change without their knowledge complicating their future managements with legal implications.

There is very limited data on the impact of identity fraud in Australia, particularly in the health care setting. According to the Department of Home Affairs [5], there were almost 100,000 incidents of identity fraud recorded by commonwealth agencies in Australia in 2014–2015 with an estimated economic cost of $2.6b per year. The Medicare card is amongst one of the most at risk of misuse, accounting for 14% of documents verified through the Document Verification Service.

Cases of identity fraud discovered during these patients’ hospital stay must be handled with care as they are likely to self-discharge upon the unveiling of their true identities. The relevant authorities should be contacted after obtaining appropriate medicolegal advice. The patients’ real identities and fraudulent aliases should be linked and updated on their health records and distributed to the relevant health centres. Health professionals must also ensure the protection of the confidentiality and safety of those who have been affected by identity fraud. Lastly, a multidisciplinary team approach to these presenting ‘patients” involving surgeons, psychiatrists, nurses and allied health staff is necessary to explore their underlying intent or psychiatric disorders to provide them with the appropriate education, treatment and follow-up care.

Potential strategies to reduce the impact of identity theft in the hospital setting include incorporating a photo identification to the Medicare Card or using multiple forms of identification when creating medical records. Finally, it is important to recognise that this is a complex issue and heightened vigilance is required amongst doctors to avoid causing harm to patients.

## Conflicts of interest

There are no conflicts of interest, financial, personal or otherwise which could influence bias.

## Sources of funding

No funding was needed for this case series.

No sponsors were involved with this case series.

## Ethical approval

Ethical approval was not required for this case series as it is exempted by our institution.

## Consent

Appropriate consent was obtained for submitting this case series for publication. Identifying details have been omitted.

## Author contribution

Dr Jason Cui – Corresponding author. Review of patient notes, drafted article. Critical analysis and approval of final submission.

Dr Rasika Hendahewa – Consultant surgeon involved in patient care and decision making. Critical analysis and approval of final submission.

## Registration of research studies

Registration completed through Research Registry at http://www.researchregistry.com.

UIN: **researchregistry4473**.

## Guarantor

Dr Jason Cui (MBBS).

General Surgical Department, Caboolture Hospital, Caboolture, QLD, 4510, Australia.

Dr Rasika Hendahewa (MBBS, FRACS).

General Surgical Department, Caboolture Hospital, Caboolture, QLD, 4510, Australia.

## Provenance and peer review

Not commissioned, externally peer reviewed.

## Study design

This is a single-centre case series that is retrospective in design.
